# Torsional wave elastography to assess the mechanical properties of the cornea

**DOI:** 10.1038/s41598-022-12151-2

**Published:** 2022-05-19

**Authors:** Jorge Torres, Inas H. Faris, Antonio Callejas, Felisa Reyes-Ortega, Juan Melchor, Miguel Gonzalez-Andrades, Guillermo Rus

**Affiliations:** 1 Ultrasonics Lab (TEP-959), Department of Structural Mechanics, University of Granada, Granada, Spain; 2 Biomechanics Group (TEC-12), Instituto de Investigación Biosanitaria, ibs.GRANADA, Granada, Spain; 3 Department of Ophthalmology, Maimonides Biomedical Research Institute of Cordoba (IMIBIC), Reina Sofia University Hospital, University of Cordoba, Cordoba, Spain; 4 Excellence Research Unit “ModelingNature” (MNat), Universidad de Granada, Granada, Spain; 5 Department of Statistics and Operations Research, University of Granada, Granada, Spain

**Keywords:** Biomedical engineering, Mechanical engineering, Diagnostic markers, Predictive markers, Prognostic markers

## Abstract

Corneal mechanical changes are believed to occur before any visible structural alterations observed during routine clinical evaluation. This study proposed developing an elastography technique based on torsional waves (TWE) adapted to the specificities of the cornea. By measuring the displacements in the propagation plane perpendicular to the axis of the emitter, the effect of guided waves in plate-like media was proven negligible. Ex vivo experiments were carried out on porcine corneal samples considering a group of control and one group of alkali burn treatment ($\hbox {NH}_\text {4}$OH) that modified the mechanical properties. Phase speed was recovered as a function of intraocular pressure (IOP), and a Kelvin-Voigt rheological model was fitted to the dispersion curves to estimate viscoelastic parameters. A comparison with uniaxial tensile testing with thin-walled assumptions was also performed. Both shear elasticity and viscosity correlated positively with IOP, being the elasticity lower and the viscosity higher for the treated group. The viscoelastic parameters ranged from 21.33 to 63.17 kPa, and from 2.82 to 5.30 Pa s, for shear elasticity and viscosity, respectively. As far as the authors know, no other investigations have studied this mechanical plane under low strain ratios, typical of dynamic elastography in corneal tissue. TWE reflected mechanical properties changes after treatment, showing a high potential for clinical diagnosis due to its rapid performance time and paving the way for future in vivo studies.

## Introduction

**Figure 1:**
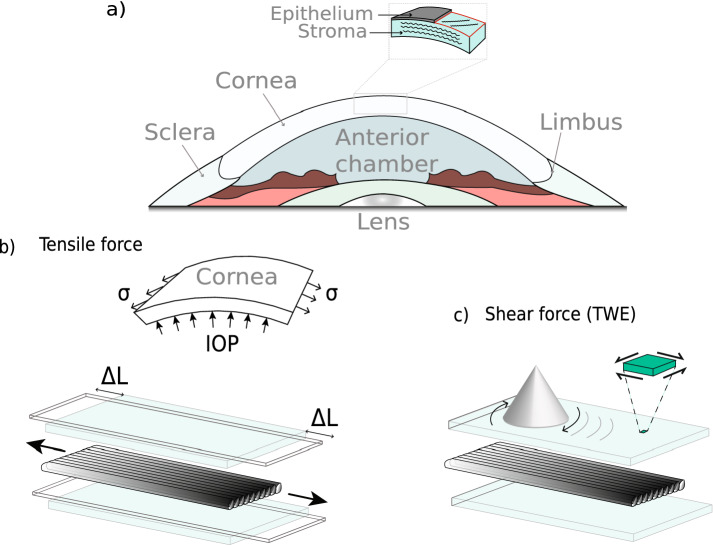
Sample description and applied loads: (**a**) Sagittal superior plane of the eye, where the stroma and stacked collagen lamellae are depicted. (**b**) In the upper part, it can be seen how the IOP creates circumferential stresses on the cornea, conferring a state of preload and therefore of nonlinearity that affects wave propagation. The mechanical response to a tensile load is shown at the bottom. (**c**) The torsional wave emitting disk generates a mechanical response to the shear-type stimulus on the anterior surface of the cornea, which is in the same plane as the stretching produced by the tensile test.

The mechanical state of the cornea is assumed to be defined almost exclusively by the stroma^1^. The rest of the layers that comprise this tissue are believed to have indirect contributions, as in the case of the endothelium, which deturgesces the cornea to maintain adequate functionality^2^. The main component to which mechanical stability is attributed is collagen, whose fibers are packed and arranged, forming stacked oriented lamellae^3^ (Fig. 1a). Significant changes in the organization of this layer may lead to corneal disorders, including progressive and generalized weakening of the cornea, corneal opacity, and subsequent vision loss^4^. Among those, chemical eye burns, keratoconus and other ectatic disorders^5,6^ emerge as a priority because of their incidence, severity, and impact on the patient’s quality of life. More than 70% of chemical burns in the eye are accidents that occur at work and 84% of them are due to alkali chemicals^7^. These high pH chemical reactants show a high penetration that can cause severe injury to external organs and tissues like the cornea^8^. They can even penetrate the anterior chamber damaging the lens, the ciliary body, and the trabecular meshwork, causing cataract and secondary glaucoma in severe cases. The damage caused depends on the exposure time to the chemical, showing different grades of severity^9^.

From a physiological perspective, the primary function of the cornea, together with the lens, is to redirect light to the retina. Whereas the lens can change its geometry to improve focus on objects near it, the cornea has a fixed focus^10^. This means that pathologies or alterations in the cornea that manifest themselves as changes at the microstructure level directly affect its refractive capacity, primarily by modifying optical power and transparency^11^. It is believed that early diagnosis of these states could be achieved by evaluating constitutive mechanical properties, since changes in these properties occur before any of the macroscopically visible structural changes observed during routine clinical evaluation^12,13^. In addition, as a viscoelastic tissue, the relevance of viscosity has been evidenced in recent studies, which adds to the scene an important contrasting parameter^14,15^. Mechanical differences between physiological and pathophysiological conditions are expected to be sufficiently relevant to be included in the diagnosis and design of treatments adapted to each patient, whose evolution could be monitored and even predicted^16^. Therefore, effective visual care might be supported by reliable quantitative mechanical techniques.

Current capabilities from state-of-the-art are focusing on the direct quantification of mechanical properties considering different principles since a multiscale approach has not yet been sufficiently developed, where changes in the microstructure are used to explain macroscale properties. The most widespread in vivo method is designed to quantify IOP by noncontact tonometry^17^. Its working principle is based on the difference in pressure between applanation events, providing two indices of viscosity and elasticity; however, its predictive power has not been well demonstrated, since the estimation combines geometric and mechanic variables^18^. It is also known that tonometry implies high displacements for a correct measurement, which leads to a nonlinear regime that is being ignored^19^. Using the main idea of passive elastography, ocular pulse elastography was recently proposed^20^. Ocular pulsation was simulated at a typical heart rate in ex vivo eye globes, and high-frequency displacement tracking was performed. As the authors discussed, speckle tracking over consecutive frames was highly affected by several involuntary eye motions, introducing noise artifacts during postprocessing. In other techniques based on static elastography^21^, a relative strain field caused by an unknown stress field could not be considered a reliable quantitative biomechanical evaluation. Encouraged by promising results in other soft tissues, several studies applied remote palpation by acoustic radiation force to obtain 2D elasticity maps^22–26^. Polarized shear waves were induced within the field of view of the transducer and then displacements or velocities were tracked at a high frame rate as the waves propagated. These approaches provided valuable information, mainly due to a fine resolution (> 15 kHz), generating high sensitivity images at the nearly micrometric level. Still, for the time being, it is difficult to reengineer a clinical setup for its in vivo implementation. Scanning time could take tens of seconds or even minutes, and the characterization and application of the high energy acoustic radiation force remain elusive, especially due to the thin corneal geometry, where complex wave patterns governed by guided waves could bias the results^27^. Lastly, optical coherence elastography (OCE) stands as the most prolific technique in terms of publications^28,29^. OCE main advantages were the microscale resolution of the images together with the microscale sensitivity in motion detection and the noncontact approach. This has prompted the development of material models that consider the complex propagation of guided waves, also including physiological conditions^30^. Even so, it was not exempted from limitations depending on the imaging modality, such as long acquisition imaging times, from several seconds to minutes, repeated stimulation that could lead to bias due to relaxation effects in the tissue, a very precise positioning where a slight motion could cause image artifacts, and a low frame rate in 2D imaging^31,32^.

Recently, the concept of torsional waves applied to elastography was introduced^33^. Preliminary work was carried out in the field of obstetrics to explore the feasibility of in vivo implementation^34^. Here we proposed developing an elastography method based on torsional waves (TWE) adapted to the specificities of the cornea. An emitting disk contacted the outermost layer and generated shear (torsional) waves that propagated axisymmetrically in depth and radially. A piezoelectric sensor adapted to the curvature of the cornea collected travelling waves through the specimen, which were used to derive biomechanical-related properties. A distinctive feature of this technique was that we obtained information about in-plane shear deformation in the cornea, therefore, the influence of guided waves, likely present in out-of-plane propagation, was found to be negligible. Measurements were performed on ex vivo porcine corneas, considering two groups, one control group and an alkali burn group that modified the mechanical properties of the cornea. After that, the corneal buttons were excised for a tensile test to compare the trends of the estimated results. Both techniques provided responses in the same mechanical plane (Fig. 1). The experimental results showed evidence that this technique could discern different mechanical states and paved the way for supporting current in vivo techniques, given its methodological simplicity and fast parameter reconstruction.

## Methods

### Ex vivo alkali burn corneal model

Porcine corneal samples were obtained from a local abattoir and enucleated immediately postmortem, then placed in phosphate buffered saline solution (PBS, pH 7.4) solution until testing to prevent moisture loss. The buffer solution was prepared using di-Sodium Hydrogen Phosphate anhydrous (Reag. Ph. Eur. 99%), Potassium di-Hydrogen Phosphate (Reag. Ph. Eur. 99% purity) and Sodium Chloride (USP, BP, Ph. Eur. JP 99%) from Panreac AppliChem. To produce changes in mechanical properties, a treatment solution associated with alkali burns that modified the structure of the stroma was selected taking into account the most frequent chemical reactants in house or industrial cleaning products^35^. Ammonium hydroxide 3 mM ($\hbox {NH}_\text {4}$OH at 10% v/v) was used at a similar concentration as usual fertilizers and the double concentration found in cleaning products^36^. Ammonium hydroxide solution (EMSURE ACS, Reag. Ph Eur 28–30%) was purchased from Merck. Treatment solutions were prepared in MilliQ water. All reactants were used as received without further purification. No treatment was applied to the control group, only washing for 1 min in PBS.

**Figure 2:**
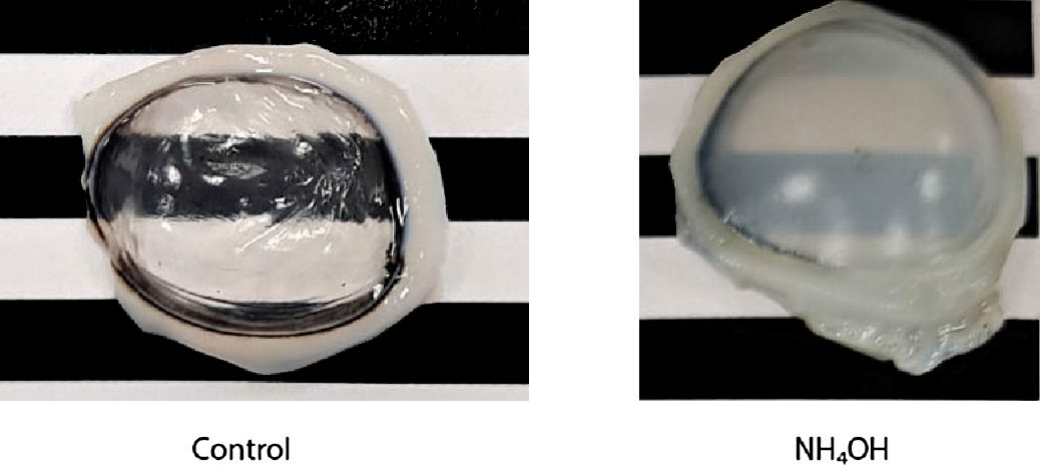
Porcine corneal samples from the control and treated groups. The background pattern evidenced structural changes that ammonium hydroxide ($\hbox {NH}_\text {4}$OH) causes.

A total of 16 samples were tested within the first 10 h after excision. They were classified considering the treatment and exposure time (Fig. 2), resulting in 2 groups with 8 samples in each of them: control and $\hbox {NH}_4$OH for 5 min. Treatment was applied to the entire eye globe by immersion in 50 mL of the respective solution during the established exposure time. The treated samples were then washed in PBS, and the epithelium was mechanically removed using a spatula. Visually, we can observe that the damaged cornea leads to the opacity of the stroma. All corneal samples were used for a subsequent examination with TWE and tensile test.

### Torsional wave elastography

An elastography device with a matching design was proposed, where the excitation and sensor parts were assembled. The excitation generated torsional waves in the specimen by direct contact. These were shear waves that propagate axisymmetrically within the cornea’s surface, transmitting an oscillatory rotation through a cone-shaped disk (4 mm base) that was driven by an electromechanical actuator. Two parallel rings formed the sensor, with four slots in each inner face, where four ceramic piezoelectric elements (PZT-5) working in shear mode were connected with a conductive resin^33^. This configuration minimized the recording of unwanted compressional waves^37,38^.

**Figure 3:**
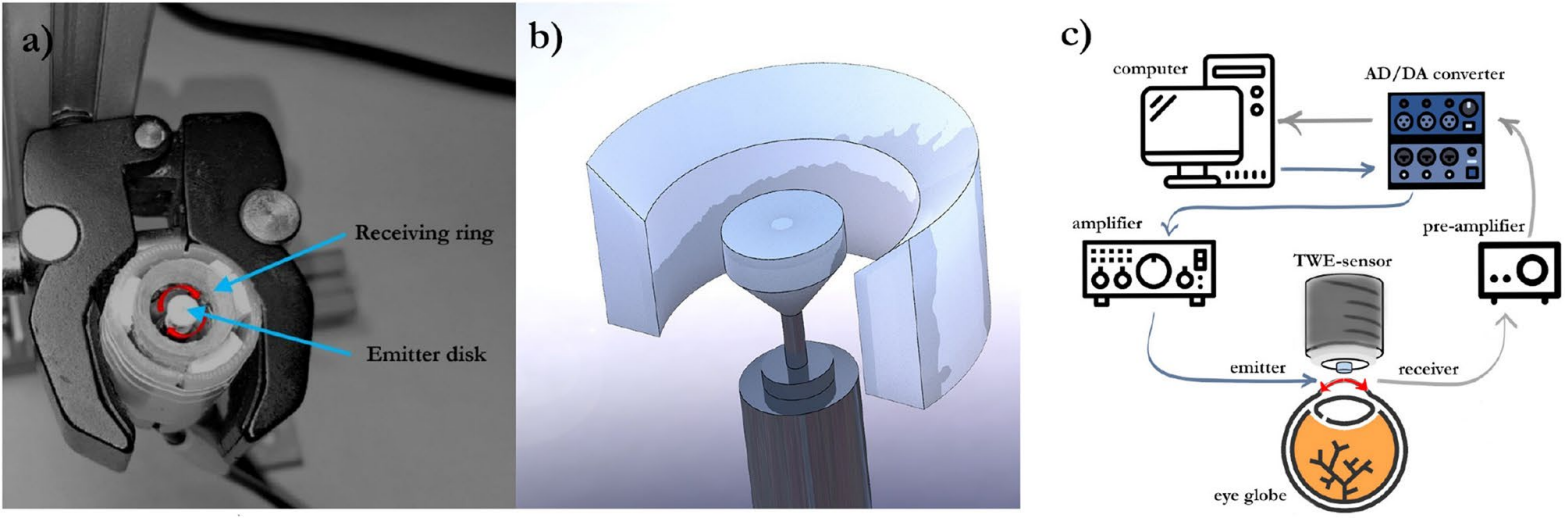
Torsional Wave Elastography (TWE): (**a**) the final assembly with the contact components marked and the attenuator elements visible in white on the contour of the device; (**b**) rendered isometric view where the curvature of the contact ring is visible; (**c**) experimental setup for corneal tissue characterization using TWE.

The dimensions and geometry of the contacting receiving ring were selected to match the samples^39^. The external and internal diameters were 13 mm and 9.6 mm, respectively, with an internal curvature that covered the corneal shape completely. This set was assembled in a casing with mechanical attenuators that also centered the emitting disk relative to the receiving ring (Fig. 3a,b). All components were 3D printed using a biocompatible photopolymer resin (MED610, Stratasys Inc., Eden Prairie, MN, USA) except for the casing that was printed in PLA (polylactic acid).

A multichannel AD/DA converter with 24 bits and a 192 kHz sampling rate was used to generate and record the received signals. In principle, this sampling frequency increased the maximum wave speed limit sensitivity compared to ultrasound elastography modalities. The digital to analog converter outputs a single sinusoidal pulse of 500, 600, 700, 800, 900, and 1000 Hz, the six frequencies used in this study, connected to a sound amplifier (100 W) that transmitted a load of 25V peak-to-peak to the emitter. Immediately after that, the recording step started and a preamplifier captured the receiver’s electrical signal (40 dB gain), to reach the AD converter; this setup is illustrated in Fig. 3c. During this transition, no interfering effect between steps was observed. A 5 kHz low-pass filter was applied to the received signal to eliminate the high-frequency jitter. To reduce random noise, the resulting signal consisted of an average of 16 signals, acquired at 200 ms time intervals, for a total measurement time of 3.2 s. Prior to measuring the sample, a calibration signal was taken to counterbalance crosstalk effects. Finally, a dedicated algorithm was used to calculate the phase shear wave speed. The cornea behaves as a dispersive medium, therefore its mechanical response depends on the excitation frequency. By leveraging this fact, the time-of-flight was computed for each excitation by subtracting a quarter of the period, corresponding to the frequency of the highest energy in the response, from the first peak. Speed was retrieved by considering the distance traveled and this time. Note that medium curvature has been shown to be irrelevant for speed calculation^40^. All elements were computer controlled using high speed communication ports and a Matlab environment (R2018b, The MathWorks Inc., Natick, MA, USA).

The entire porcine eyeball was placed in a custom-made holder and the cornea was oriented side up. The device covered the cornea with gentle pressure, a fixed force of 500 mN, and all samples were measured three times by repositioning for averaging. The eyeball was cannulated with a needle near the limbus to access the anterior chamber. This needle was connected to a saline infusion reservoir, which was used to modulate IOP by adjusting its height. Measurements were performed at pathophysiologically relevant controlled pressures of 5, 10, 15, 20, 25, and 30 mmHg, waiting 2 min for IOP stabilization.

Preliminary safety considerations were examined. In this modality, no cavitation-related problems were expected. Induced displacements were measured with an ultrafast ultrasound scanner (Vantage 256, Verasonics Inc., Redmond, WA, USA) that tracked the wave propagation with plane waves at a rate of 25 kHz, placing a 18.5 MHz transducer in a plane perpendicular to the axis of the emitter in the same setup previously described. The displacement peaked in the control group at around $2\,\upmu \text {m}$, thus a linear regime was assumed. In a recent study with a similar configuration^34^, the maximum acoustic intensity of TWE was estimated at 5.3 mW/$\hbox {cm}^2$, well below the spatial peak temporal-average intensity limit of 17 mW/$\hbox {cm}^2$ set by the FDA for ophthalmic applications^41^.

To characterize the viscoelasticity of the tissue given the dispersion curve, a rheological model is the most straightforward approach. The diligent task of identifying the best model is currently under discussion in the scientific community. The Kelvin–Voigt model (KV) was chosen in this work due to its widespread use in the literature and the low range of frequencies^28^. This model is composed of two elements that connect the physical interpretation with the experimental values. Shear elasticity and viscosity were obtained by fitting the dispersion curve using a nonlinear least squares (BFGS) method.

### Torsional wave dispersion in confined media

The configuration of the TWE device was optimized to emit and receive displacements in the same plane of the emitter axis, which in the anterior surface of the cornea corresponds to the lamellae plane or the in-plane mechanical response, see Fig. 1c. To study the effects introduced by confined geometries on the propagation plane perpendicular to the axis of the emitter (axysimmetrical propagation), two prismatic phantoms were manufactured. Isotropic viscoelastic polyacrylamide phantoms (PAAm) have been manufactured with 50% concentration following the recipe of Kumar et al.^42^. This material provided a surface similar to the cornea, ideal for our contact approach, and reached speeds in the range of the corneal tissue. The phantoms were made with different thicknesses, thin ones of 1.2 mm mimicking, a typical thickness of a porcine cornea, and bulk ones of 15 mm thickness. The phantoms were measured three times with TWE at constant pressure to average, and an ultrasound gel was placed underneath to simulate aqueous humor.

For the purpose of validation, the induced displacements were measured with the previously described configuration of the Verasonics research equipment. The L22-14v transducer was used to receive torsional waves generated by an emitter identical to the one encapsulated in our torsion wave probe. The phase speed was retrieved with the classical method of computing the phase from the Fourier transform and fitting a linear model to the propagation distance at each frequency.

### Tensile test setup

As a reference to TWE, uniaxial tensile tests were conducted to obtain the shear wave speed of porcine corneas in the two studied groups. The device used was ElectroForce 3200 Series (TA Instruments, EEUU). The setup consisted of two clamps with specific surface roughness to avoid sample slippage. The lower clamp was attached to a load cell whose maximum capacity was 22 N, with a resolution of 0.001 N.

After corneal button extraction, all samples were cut into vertical strips in the nasotemporal direction to avoid orientation dependency and without the sclera. The samples were gripped by two shoulders and the main deformation occurred in the central cross section (gauge section). The dimensions of the samples (length, width, and thickness) were measured using an electronic caliper. The average thickness was $1.32 \pm 0.0608$ mm and $1.87 \pm 0.1277$ mm for the control and $\hbox {NH}_4$OH groups, respectively.

The samples were preconditioned with one load/unload sequence at 1 N to reach a stage of stable behavior^43^. Then the tissues underwent another load/unload quasi-static uniaxial sequence at a rate of 0.05 N/s up to a limit force of 2 N. The test was performed by moving the lower clamp through an extended stroke accessory. The corneal samples were kept continuously hydrated by spraying them with PBS to prevent severe alteration of the mechanical properties during the experiment. To obtain the complete stress–strain curve, the stress was obtained by dividing the force measured in each increment of displacement by the initial section in the most unfavorable area, and the deformation by dividing the displacement by the initial length between the clamps. This initial length was set when the sample was stretched under a load of 0.01 N. The thickness of the samples was considered constant throughout the test (Fig. 5).

Following the nonlinear behavior of the stress-strain curve, we approximated the stress state caused by the internal applied pressure during TWE measurements for a valid comparison. The curve was analyzed in six regions corresponding to the stress associated with each of the previously imposed IOPs in the cornea. This circumferential stress (see Fig. 1b), under the thin-walled hypothesis, was calculated according to the following equation,

1 $$\begin{aligned} \sigma =\frac{P \cdot r}{2 \cdot e}, \end{aligned}$$

where *P* was the internal pressure, *r* the mean corneal radius (8.45 mm)^44^, and *e* the average cornea thickness. To determine the Young’s modulus, a searching algorithm was implemented in Matlab to identify the strain region that corresponded to the circumferential stress for each IOP. Then with assumptions of incompressibility, linearity, and isotropy, the shear wave speed was retrieved.

## Results

### Dispersion analysis with material thickness

**Figure 4:**
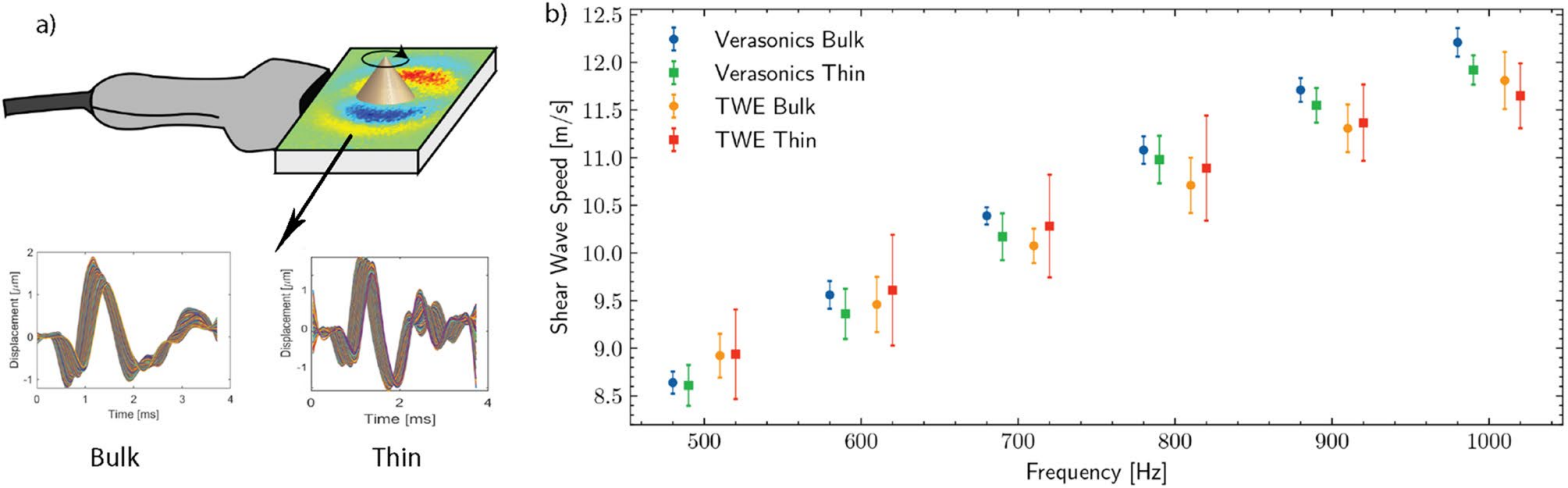
Exploring the torsional wave propagation under different geometries: (**a**) Shear wave imaging was performed on the horizontal plane of both thin and bulk polyacrylamide phantoms. The time profile of the displacements was similar for both geometries. (**b**) Comparison plot of TWE (torsional wave elastography) and verasonics (shear wave imaging) results. Verasonics validates both the values obtained and the negligible effect of guided waves. Results are displayed as mean ± standard deviation.

Figure 4a shows similar displacement in time profiles for both geometries: thin and thick polyacrylamide phantoms, with the difference of some ripples at the end of the thin phantom that were not relevant for analysis. Figure 4b displays a dispersion curve for a range of excitation frequencies 500–1000 Hz for both methods and geometries. We noticed that Verasonics, as the gold standard, validated our proposed device and that the confined geometries did not affect our wave propagation analysis. The effect of guided waves, if present under this excitation, was neglected in this measurement plane.

### Tensile tests at low strains

**Figure 5:**
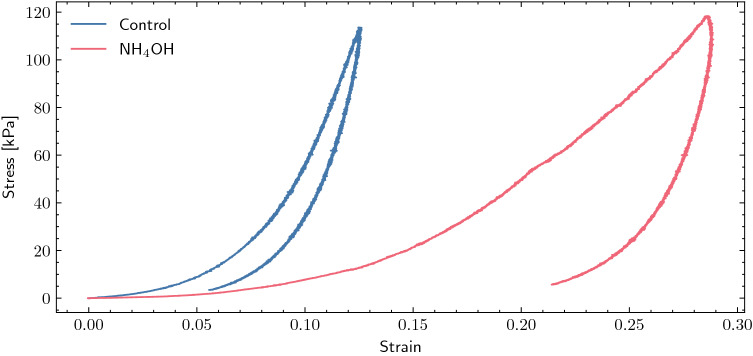
Typical load/unload curves for control and $\hbox {NH}_4$OH samples. Hysteresis is more pronounced after chemical treatment. The limit force was 2 N.

Figure 5 shows the representative cyclic behavior of the studied groups, where the maximum stress value, relative to 2 N, marks the starting point of the unloading phase.

By observing Fig. 5, the control group (blue line) has a steeper slope with respect to the treated group (red line). Despite the 2 N limit, different stresses were reached in each sample due to different cross sections. The circumferential stresses ranged from 8.71 to 13.27 kPa for both groups, and the corresponding strain ranges in the curve were 4–6% for control, and 11–13% for $\hbox {NH}_4$OH. Additionally, it can be observed how the enclosed area (hysteresis) varies in each cycle, increasing in the case of the treated sample compared to the control sample.

**Figure 6:**
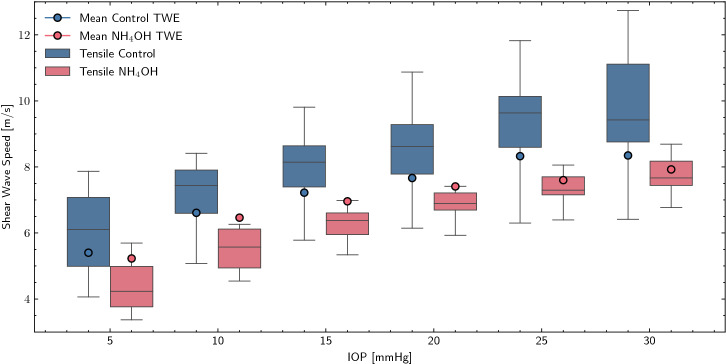
Comparison of shear wave speed from uniaxial tensile tests and TWE (torsional wave elastography) results for each intraocular pressure (IOP). The boxplot of the tensile tests shows the same trend as the mean shear wave speed of TWE (blue points—control samples, red points—$\hbox {NH}_4$OH samples).

Figure 6 shows a comparison of the shear wave speed of the uniaxial tensile test and TWE for both groups (control and $\hbox {NH}_4$OH samples) and for each IOP. TWE results are displayed as the mean of all frequencies to verify that both techniques follow the same trend. We observed that strain varied nonlinearly with IOP and shear wave speed increased as well for both techniques. Furthermore, the values of the treated group were lower than those of the control sample.

### Corneal viscoelastic characterization by TWE

**Figure 7:**
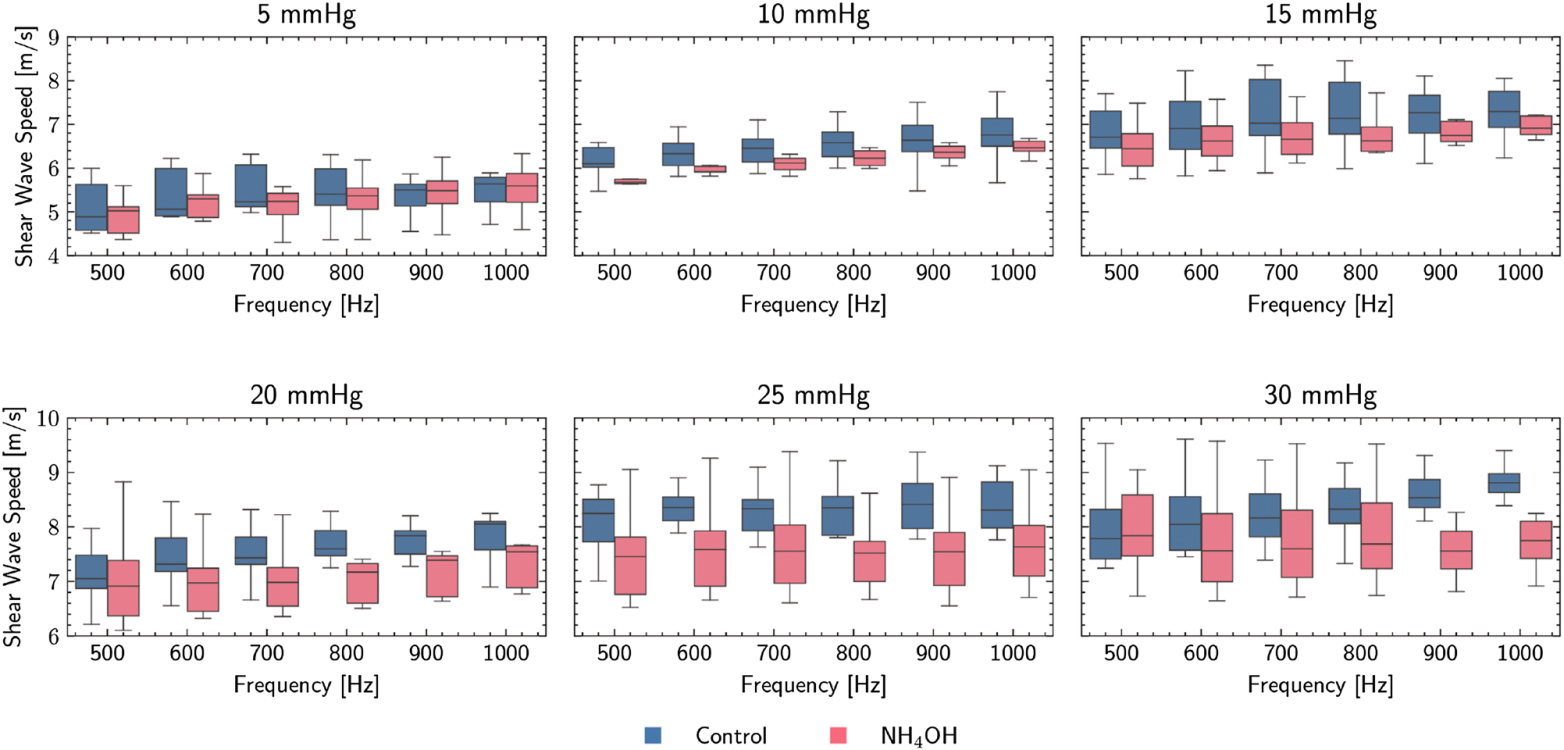
Torsional wave elastography (TWE) results: box plots of TWE dispersion curves differentiating each group. Two increasing trends are observed, the first with increasing frequency, and the second with increasing intraocular pressure (IOP). Ammonium hydroxide ($\hbox {NH}_\text {4}$OH) group exhibits lower values than the control group.

Figure 7 displays a summary of the dispersion curves obtained by TWE for each group under different IOPs. The speed of the shear wave increased with frequency and IOP for both the control and $\hbox {NH}_4$ OH groups, showing lower values for the treated group. It can also be seen in all results figures that the increase in IOP accentuates the differences between the groups studied.

**Figure 8:**
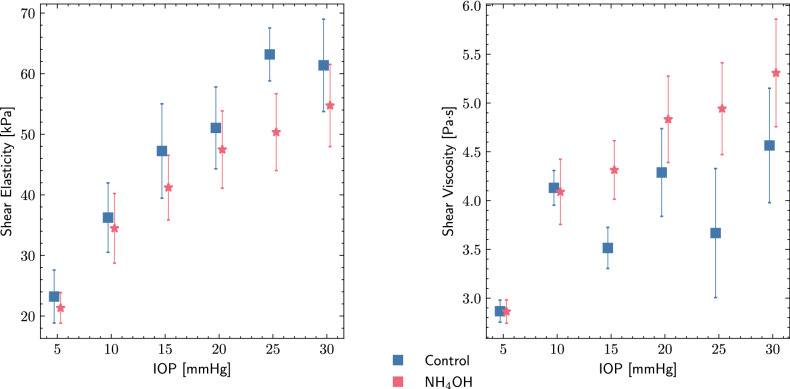
Viscoelastic parameters for ex vivo porcine corneal samples using Kelvin Voigt (KV) model on elastography results. Shear elasticity and viscosity are displayed for Ammonium hydroxide ($\hbox {NH}_\text {4}$OH) and control groups under different intraocular pressures (IOP). The results are displayed as mean ± standard deviation.

As a dispersive medium, the cornea showed that different excitation frequencies affected the final estimation, with higher speed related to higher frequencies. Figure 8 shows the Kelvin Voigt (KV) fitted values of the dispersion curves of both control and $\hbox {NH}_4$OH groups for each IOP value. Shear elasticity increased with IOP in both groups, being higher in the control group. The viscosity was higher in the treated groups; this agreed with the results shown in Fig. 6.

## Discussion

### Plane of measurement and propagation

In this study, the diagnostic capabilities of TWE were reported on a geometry as specific as that of the cornea. Figure 4 reveals that when displacements were measured in the propagation plane perpendicular to the axis of the emitter, there were no differences in speeds at different thicknesses. This meant that by measuring the direct wave, the results were not affected by guided waves in the corneal thickness. The use of the torsional wave technique reduced assumptions of complex wave models. When soft tissue is deformed at low ratios, typical of dynamic elastography, collagen bends easily and offers little resistance; the response is taken over by other stromal components and interlamellar forces^45^. As the deformation increases, the collagen fibers begin to align with the load, inducing greater stress and thus higher elastic modulus. These strain ranges (> 20%) are those shown in various studies (see Fig. 6 in Pitre et al.^40^), resulting in values in the MPa range. For our part, we leveraged the resolution of the load cell and the 10 kHz data acquisition rate of the software to generate curves with sufficient resolution in the strain range 1–10% to obtain results in kPa magnitudes. This allowed a direct comparison with tensile tests and a consistent mechanical validation of the proposed technique (Fig. 6). We noticed that with our methodology, the tensile results were similar to TWE, so that the geometrical constraints imposed, such as clamping and loss of curvature had minimal effect at such low strains. However, the determination of circumferential stress due to IOP needs to be carefully interpreted, since we assumed the cornea to be spherical and its thickness to be uniform.

It is important to emphasize that all measurements were performed on collagen lamellae planes (in-plane), unlike the ultrasound and OCE techniques^40^ presented in the literature, which measured out-of-plane propagation. Studies that considered that guided waves were created within the corneal tissue concluded that the use of Lamb wave models was necessary to provide a more accurate description of the viscoelasticity of the cornea^24,46^. We observed that their results were below 10 kPa, which was supposed to reflect the out-of-plane (interlamelar shearing) shear modulus. When no Lamb wave model was considered, several studies reported a shear modulus between 40 and 80 kPa^14,23,25,47,48^. Our results of shear elasticity ranged from 21.33 to 63.17 kPa for the cornea in the lamellae plane (in-plane). Assuming homogeneity and isotropy, these values were consistent with the previous ones, even though to our knowledge, none of them have evaluated a rheological model in this propagation plane. Although different planes of mechanical behavior have been studied, it would be interesting to elucidate which of them could be more sensitive to pathological-related changes.

This evidences that our current knowledge of the role of viscoelasticity in corneal health status is limited. The procedures presented in the literature are still in the research phase, besides obtaining a dispersion curve is a complex analysis of the signal that consumes considerable time and is not integrated into clinical equipment focused on optics. In the specific case of Verasonics, which is research-only equipment, the FDA has not authorized them for clinical in vivo application. The proposed TWE technique presented values as an average of global mechanical properties that translated into a fast reconstruction method, with hardly any postprocessing. The Kelvin–Voigt parameters reconstruction took around 3 s in situ. A significant advantage was the low energy deposited in the medium compared to ARF-based techniques. Here we have used six frequencies, observing a lower variability at higher frequencies. This flexibility in choosing the excitation frequency within the limits of the tissue response could be used to improve the SNR or explore the potential for attenuation and dispersion in specific ranges. Unlike other techniques, where a map was reconstructed, no analysis artifacts were detected or included, such as diffraction or guided waves.

### Alkali burn Corneal model effects on viscoelasticity

Several studies have tried to shed some light on the potential of viscoelastic biomarkers by associating corneal hysteresis with viscosity in air puff applanation experiments, since they observed lower hysteresis values in patients with keratoconus, post LASIK, and Fuch’s dystrophy^49^. In the literature, there is a lack of corneal shear viscosity values under the same configuration for comparative purposes. Shear viscosity or viscosity parameter was mainly reported after using OCE, with values below 1 Pa s^14,15,46,50^, whereas this study reported a range of 2.82 to 5.30 Pa s. The increasing viscosity was expected since it was directly related to the increasing shear wave speed inherent to the KV model. In this measurement plane, the dispersive effects could be attributed to the viscosity of the tissue and not to the guiding of waves as previously discussed.

The variability of results was a consequence of the different timescales and lengthscales of each technique, wave models, and experimental conditions, reaching ambiguous conclusions. In this study, a simple Kelvin–Voigt model was used. The application of this model was subject to the assumption that the medium was macroscopically homogeneous, isotropic, and linear, besides no guided waves were generated.

Both shear elasticity and viscosity were altered after chemical treatment. In the alkali burn group ($\hbox {NH}_4$OH), the speeds and elasticity parameters were lower than in the control group. This loss was attributed to the disruption of lamellae, interweaving and collagen cross-linking in a comparable manner to keratoconus^51^. As for shear viscosity we believed that the high values were due to the highly unusual thickness of the corneas, since we could not found any other study with such values. Besides, we used ultrasonography and confirmed these thicknesses. As opposed to elasticity, the viscosity was higher for the treated group, which agreed with its hysteresis curve in Fig. 5, representing a greater loss of energy, meaning that its viscous damping was more pronounced. Its reaction to the chemical treatment was to increase the corneal thickness in about 30%, probably in response to an inflammatory process. Another factor essential during ex vivo experiments, which we controlled, was hydration. It is known to be a confounding factor due to corneal dehydration, resulting in tissue thinning^2,52^. Since the epithelium was removed, water could easily penetrate the tissue and swell it.

### Correlation of shear elasticity and shear wave speed with IOP

The effect on wave speed and viscoelasticity due to varying tissue stress state was studied by using different IOPs^53^. As in other investigations^24,54^, we found a positive correlation with speed and elasticity. As IOP increased, the variability of the measurements increased. Noteworthy are the results for an IOP of 30 mmHg, whose values did not differ much from those of 25 mmHg. This is believed to be due to complex wave propagation in which linear models are no longer valid^55^. Very recently, differences have been reported when IOP was considered during OCE measurements, suggesting that changes in mechanical properties and IOP were independent and could reveal different health states^30^. However, Ramier et al.^47^ found no correlation between these magnitudes, possibly due to a convenient distribution of stress in the human cornea in vivo. Interestingly, shear viscosity was positively correlated with IOP. We hypothesized that IOP-induced stress compressed the stroma, compacting all its components^56^. This derived in an alteration of the viscous proteoglycans and shear interactions with the extracellular matrix.

### Limitations and future studies

Some limitations of the current method need to be detailed. We likely missed focal abnormalities, since no 2D image was obtained. Boundary conditions like the stress distribution generated by IOP conferred a preloaded state, which could be modified because of the direct contact. The physiologic conditions surrounding biomechanical quantification should be included to correct for possible bias. IOP could be measured with tonometry, whose magnitude will define the calculated mechanical parameters. Thus, TWE has the potential to be integrated into conventional examination procedures to achieve a more direct interpretation of the experimental parameters, but several points need to be studied further. Although direct corneal contact is necessary, it is not an unfamiliar methodology with routine protocols such as tonometry or corneal pachymetry, where topical anesthetic is instilled. Recent studies have shown that this approach is feasible^46^, and that contact could be made through lenses^57^. Besides, a stable localized excitation was achieved in this modality, and by changing the diameter of the emitting disk, a shorter temporal response with increased exploitable frequency bandwidth would be expected. To further reduce contact, the receiving ring could be substituted by a small sector. To be on the side of safety, the effect of the pressure exerted when measuring will be studied in the future, both to assure an efficient propagation of torsional waves, confirming that local induced stresses do not severely affect wave propagation, and to avoid patient discomfort. The effect of sensor pressure and friction will be monitored to avoid epithelial disruption. On the other hand, this applied pressure could become an opportunity to obtain nonlinear elasticity parameters, as in acoustoelasticity, whose relevance could be substantial^35,45^. Since it is difficult for patients to maintain a fixed position or avoid involuntary movements, the short times required to take measurements are an additional benefit that helps reduce motion artifacts. Some studies have shown that the cornea is an anisotropic tissue^23,30,40^. Future studies of TWE could incorporate anisotropy and nonlinearity into the analysis and interpretation of the measurements. Additionally, one can take advantage of the axisymmetric propagation of TWE together with a sectorized ring to obtain information in several directions at the same time as a means of assessing any regional heterogeneity in cornea mechanical properties.
